# One-Year Follow-Up in a Phase 1/2a Clinical Trial of an Allogeneic RPE Cell Bioengineered Implant for Advanced Dry Age-Related Macular Degeneration

**DOI:** 10.1167/tvst.10.10.13

**Published:** 2021-10-06

**Authors:** Amir H. Kashani, Jane S. Lebkowski, Firas M. Rahhal, Robert L. Avery, Hani Salehi-Had, Sanford Chen, Clement Chan, Neal Palejwala, April Ingram, Wei Dang, Chih-Min Lin, Debbie Mitra, Britney O. Pennington, Cassidy Hinman, Mohamed A. Faynus, Jeffrey K. Bailey, Sukriti Mohan, Narsing Rao, Lincoln V. Johnson, Dennis O. Clegg, David R. Hinton, Mark S. Humayun

**Affiliations:** 1 Wilmer Eye Institute, Johns Hopkins School of Medicine, Johns Hopkins University, Baltimore, MD, USA; 2 Regenerative Patch Technologies, Menlo Park, CA, USA; 3 Retina-Vitreous Associates Medical Group, Beverly Hills, CA, USA; 4 California Retina Consultants, Santa Barbara, CA, USA; 5 Atlantis Eye Care, Huntington Beach, CA, USA; 6 Orange County Retina Medical Group, Santa Ana, CA, USA; 7 Southern California Desert Retina Consultants, Palm Desert, CA, USA; 8 Retinal Consultants of Arizona, Retinal Research Institute LLC, Phoenix, AZ, USA; 9 Center for Biomedicine and Genetics, Beckman Research Institute of City of Hope, Duarte, CA, USA; 10 USC Roski Eye Institute, USC Ginsburg Institute for Biomedical Therapeutics and Department of Ophthalmology, Keck School of Medicine, University of Southern California, Los Angeles, CA, USA; 11 Center for Stem Cell Biology and Engineering, Neuroscience Research Institute, University of California, Santa Barbara, CA, USA; 12 Department of Pathology, Keck School of Medicine, University of Southern California, Los Angeles, CA, USA; 13 Department of Biomedical Engineering, University of Southern California, Los Angeles, CA, USA

**Keywords:** implant, RPE cells, clinical trial, geographic atrophy

## Abstract

**Purpose:**

To report 1-year follow-up of a phase 1/2a clinical trial testing a composite subretinal implant having polarized human embryonic stem cell (hESC)-derived retinal pigment epithelium (RPE) cells on an ultrathin parylene substrate in subjects with advanced non-neovascular age-related macular degeneration (NNAMD)

**Methods:**

The phase 1/2a clinical trial included 16 subjects in two cohorts. The main endpoint was safety assessed at 365 days using ophthalmic and systemic exams. Pseudophakic subjects with geographic atrophy (GA) and severe vision loss were eligible. Low-dose tacrolimus immunosuppression was utilized for 68 days in the peri-implantation period. The implant was delivered to the worst seeing eye with a custom subretinal insertion device in an outpatient setting. A data safety monitoring committee reviewed all results.

**Results:**

The treated eyes of all subjects were legally blind with a baseline best-corrected visual acuity (BCVA) of ≤ 20/200. There were no unexpected serious adverse events. Four subjects in cohort 1 had serious ocular adverse events, including retinal hemorrhage, edema, focal retinal detachment, or RPE detachment, which was mitigated in cohort 2 using improved hemostasis during surgery. Although this study was not powered to assess efficacy, treated eyes from four subjects showed an increased BCVA of >5 letters (6–13 letters). A larger proportion of treated eyes experienced a >5-letter gain when compared with the untreated eye (27% vs. 7%; *P* = not significant) and a larger proportion of nonimplanted eyes demonstrated a >5-letter loss (47% vs. 33%; *P* = not significant).

**Conclusions:**

Outpatient delivery of the implant can be performed routinely. At 1 year, the implant is safe and well tolerated in subjects with advanced dry AMD.

**Translational Relevance:**

This work describes the first clinical trial, to our knowledge, of a novel implant for advanced dry AMD.

## Introduction

Non-neovascular age-related macular degeneration (NNAMD) is associated with chronic progressive atrophy of the retinal pigment epithelium (RPE) and loss of central vision. It is a major unmet medical need that impacts millions of people in the Western world.^1^^–^^3^ There are no approved pharmacologic treatments that significantly impact the progression of NNAMD, although vitamin supplementation and the Age-Related Eye Disease Study formulation have been shown to modestly delay progression.^4^^,^^5^ Although the exact pathology underlying NNAMD disease is unclear, vision loss is highly correlated with loss of the RPE in a pattern of geographic atrophy (GA). Similarly, selective loss of photoreceptor cells in other retinal degenerative diseases, such as retinitis pigmentosa, and ganglion cells in glaucoma are highly correlated with vision loss.^2^^,^^6^ Therefore, cell-based replacement strategies hold significant promise for treatment of retinal degenerative diseases, especially when atrophy of retinal tissue initiates. Several challenges have prevented cell replacement strategies from successful implementation, including identification of the optimal configuration for replacement (cell suspension vs. scaffold-supported cells), development of feasible and uncomplicated surgical delivery methods, sourcing and cGMP production of cells to meet clinical needs, methods ensuring long-term survival of implanted cells, assessment of immune responses to allogeneic tissue, and, ultimately, confirmation of normal function of donor cells in diseased host tissue.^2^

In subjects with NNAMD and GA, macular translocation surgery,^7^^,^^8^ replacement of atrophic RPE with autologous adult RPE,^9^^–^^11^ induced pluripotent stem cell–derived RPE,^12^ or human embryonic stem cell (hESC)-derived RPE^13^^–^^15^ are potentially feasible approaches to preserving or even improving vision. However, the long-term safety, survival, function and immunogenicity of these treatments are unknown. We conducted a phase 1/2a, prospective, single-arm, interventional study using a composite implant consisting of a monolayer of hESC-RPE cultured on a bioinert parylene membrane^14^ with diffusional properties that mimic those of the Bruch's membrane to replace atrophic RPE in subjects with advanced NNAMD, GA, and severe vision loss. The preliminary results from the first five subjects^14^ and the detailed surgical methods for all implanted subjects were published recently.^16^ We present here the results of the predefined safety and preliminary efficacy endpoints at 1 year for all subjects in the study.

## Methods

### Study Design

The study design^14^ and surgical methods^16^ have been described previously in detail. Institutional Review Board approval was obtained from the University of Southern California, as well as the Western Institutional Review Board, for all participating sites. Informed consent for harvesting the treated and fellow eyes from subjects in the event of death was obtained from all subjects. Clearance of an Investigational New Drug (IND) application was obtained from the Food and Drug Administration for a single-arm, prospective, phase 1/2a study to recruit and enroll up to 20 subjects to assess the safety and potential efficacy of the investigational implant called California Project to Cure Blindness Retinal Pigment Epithelium (CPCB-RPE1). The study was registered in the ClinicalTrials.gov database (NCT02590692) before enrollment was initiated. CPCB-RPE1 refers to the composite implant, including the synthetic parylene substrate and the adherent and polarized monolayer of hESC-RPE. The IND application also included a custom-designed and manufactured 19-gauge surgical insertion forceps for delivery of the CPCB-RPE1 implant into the subretinal space.

All surgeries were conducted at the Outpatient Surgery Center of the University of Southern California, Keck School of Medicine, in Los Angeles, CA. A data monitoring and safety committee was assigned for the study and reviewed all results and adverse events. The primary outcome measure of the study was safety, as assessed by clinical examination at 365 days after implantation. Predetermined secondary and exploratory endpoints included efficacy as assessed by visual acuity and visual function measures, including microperimetry. The stopping rules for the study were defined in the clinical protocol and included (1) development of an expanding mass; (2) accelerated loss of visual acuity in the implanted eye; (3) development of any serious adverse pathology associated with the delivery, immunosuppression, or use of the implant that warrants enucleation of the eye; and (4) surgical delivery-related events involving the device, implant, or surgical procedure that lead to failure of the implant delivery.

Key inclusion criteria for subjects were previously described^14^ and included age ranging from 55 to 85 years and history of advanced NNAMD, GA, pseudophakia, and severe vision loss with best-corrected visual acuity (BCVA) of ≤20/200 for cohort 1 and 20/80 to 20/400 for cohort 2. Study criteria mandated that the study eye be the worse-seeing eye. Subjects with a history of any other vision-threatening disease, including neovascular age-related macular degeneration or health conditions that would prevent general anesthesia were excluded from the study. Other key exclusion criteria included history of active malignancy within the previous 5 years, history of enrollment in another clinical trial within the previous 3 months, history of active or untreated infectious disease, or any history of immune suppression or dysfunction. A detailed list of inclusion and exclusion criteria is provided on ClinicalTrials.gov (NCT02590692).

### CPCB-RPE1 Surgery and Immunosuppression

**Figure 1.**
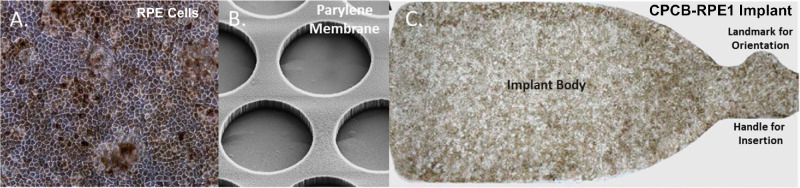
The CPCB-RPE1 implant. (A) The hESC-derived RPE cells seeded and cultured on the synthetic parylene membrane grow as a uniform monolayer of hexagonal, pigmented cells with significant similarity to RPE cells in vivo. (B) Scanning electron micrograph of the underside of the parylene membrane showing submicron-thick, circular regions (40-µm diameter) that facilitate diffusion of macromolecules and the supporting matrix. (C) Bright-field image of a single CPCB-RPE1 implant with pigmented, mature, and confluent RPE. The implant is 3.5 × 6.25 × 0.006 mm in size.

Detailed surgical methods for all subjects were described in an earlier publication.^16^ A brief summary is provided here for convenience. CPCB-RPE1 is a composite implant (dimensions 3.5 × 6.25 × 0.006 mm) that consists of a monolayer of hESC-RPE cells that are seeded and grown on an ultrathin parylene substrate as previously described (Fig. 1).^14^ Approximately 100,000 mature, polarized, and pigmented hESC-RPE cells are present on each CPCB-RPE1 implant. Subjects underwent surgical implantation of a single CPCB-RPE1 implant on study day 0. Each enrolled subject received immunosuppression using 0.075 mg/kg/day of Tacrolimus (Astellas Pharma US, Inc., Northbrook, IL) from day –8 to day 42 to achieve a therapeutic trough range of 3 to 10 ng/mL. Tacrolimus dose tapering began at day 42 and was completed by day 60. Subjects received a single intravenous injection of 250 mg methylprednisolone sodium succinate (Solumedrol; Pfizer, New York, NY) on day 0 immediately before surgery (Supplementary Fig. S1). The CPCB-RPE1 implant was manufactured and delivered to the operating suite directly from a Current Good Manufacturing Practices (cGMP) facility and within a 12-hour window preceding surgery. All subjects underwent a single outpatient pars plana vitrectomy procedure on day 0 with a constellation vitrectomy system (Alcon, Fort Worth, TX) with 23-gauge instrumentation. An OPMI Lumera 700 operating microscope with noncontact ReSight viewing system (Carl Zeiss Meditec, Dublin, CA) or a ReScan 700 operating microscope with intraoperative optical coherence tomography (iOCT; Carl Zeiss Meditec) was used as indicated in the Results section. In all cases, the CPCB-RPE1 implant was inserted with the use of the custom insertion forceps described above and published previously.^14^^,^^16^ Perfluorocarbon heavy liquid (PFO; Alcon) was instilled to flatten the macula. In all cases, the eyes were filled with 5000cS silicone oil for long-term tamponade.

### Postoperative Clinical Evaluations and Retinal Imaging

Details of the clinical evaluations and structural and functional imaging methods have been described previously and are summarized here for convenience.^14^^,^^16^ A timeline of study visits is provided in Supplementary Figure S1. Postoperative clinical evaluations and imaging were performed by study principal investigators and staff that were not involved in the surgery or preoperative surgical evaluation. OCT was performed using a commercially available SPECTRALIS system (Heidelberg Engineering, Heidelberg, Germany). Fundus photographs were acquired on standard, commercially available color fundus cameras.

Fixation stability was performed on all subjects using the Nidek MP1 microperimeter (Nidek Technologies, Padova, Italy) at baseline and at day 365 and analyzed as previously reported.^14^ Individual fixation values that were more than 3 standard deviations (SDs) from the group mean were treated as outliers. Fixation stability for the central 2° and 4° fields was assessed as the average percent of fixation events that were contained in the respective area surrounding the preferred retinal locus from two separate trials. Microperimetric sensitivity testing was performed and recorded after fixation testing if it could be reliably performed within a 30-minute window. Due to poor baseline vision, none of the subjects in the study could complete sensitivity testing within the requisite time interval, so the requirement for sensitivity testing was eliminated to avoid subject fatigue and possible drop-out from the study.

## Results

Sixteen subjects were enrolled in the study, and 15 were implanted with the CPCB-RPE1 implant, with complete follow-up for at least 365 days. One enrolled subject was not implanted, as previously described.^14^^,^^16^ Despite not receiving an implant, this subject was followed for 1 year for safety (see below). All subjects underwent a single, outpatient surgical implantation procedure.^14^^,^^16^ No subject withdrew from the study. The median age of the subjects was 78 years (range, 69–85). Table 1 summarizes the demographics of the 15 implanted subjects at baseline.

**Table 1.** Subject Demographics

	Subject	Implanted		
	ID	Eye	Age (yr)	Sex
Cohort 1 (*n* = 6)	204	OS	85	F
	125	OS	84	F
	303	OS	84	M
	128	OS	69	F
	304	OS	82	M
	305	OD	69	M
Cohort 2 (*n* = 9)	501	OS	78	F
	130	OS	78	F
	401	OS	78	F
	403	OD	80	F
	216	OS	77	F
	404	OS	73	M
	606	OD	70	M
	502	OS	77	M
	607	OS	76	F

OD, right eye; OS, left eye.

### Baseline Examination Findings

Preoperative clinical examination and fundus photographs demonstrated that all implanted subjects had moderate to large areas of GA involving the fovea (median, 13.8 mm^2^; range, 6.0–46.4 mm^2^).^16^ The mean and median baseline BCVA values in the treated eye were logMAR 1.28 ± 0.42 (SD) and 1.20 (Q1, 1.04; Q3, 1.30), respectively. Baseline BCVA for the treated eye ranged from 20/200 to count fingers. All enrolled subjects were pseudophakic. All subjects were typed for 16 different human leukocyte antigen (HLA) class I and II alleles using high-resolution analysis. The donor allogeneic cells on the implant were mismatched at multiple HLA class I and II alleles, with the best matched pair having matches at only seven of 16 assessed alleles (manuscript submitted).

### Postoperative Examination Findings

**Figure 2.**
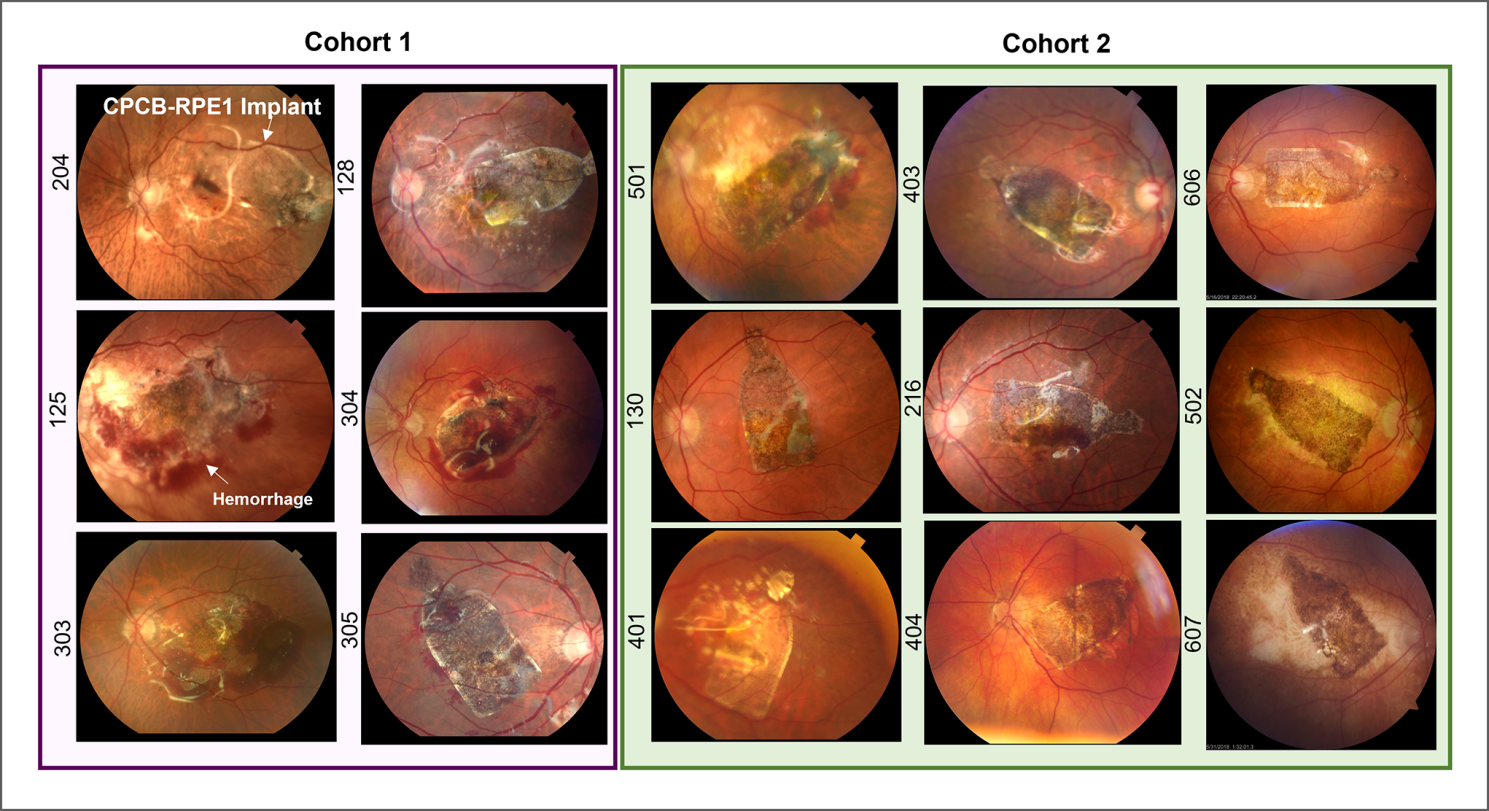
Color fundus photographs of all implanted subjects 28 days post-implantation. The *left panel* (*purple box*) illustrates all subjects enrolled in cohort 1. The *right panel* (*green box*) illustrates all subjects enrolled in cohort 2. Numbers on the left of each image denote the subject identifier. In all cases, the CPCB-RPE1 implant was successfully targeted to the subretinal space including the area of geographic atrophy and covering the majority of the lesion. Hemorrhage at day 28 was noted in four (subjects 125, 303, 304, and 305) of the six implanted subjects in cohort 1 and was reduced substantially at the same post-implantation time point in cohort 2.

Intraoperative results have been previously described and have demonstrated that all implants were placed consistently in the subretinal space and were reliably targeted to the area of GA, with the percentage of GA covered (median, 87%; range, 31%–100%) dependent on the baseline size of its area.^16^ Postoperative evaluations demonstrated no evidence of implant migration in the subretinal space. Fundus photographs of all implanted subjects at 28 days post-implantation are provided in Figure 2. Subretinal hemorrhage was noted in four of the first six implanted subjects (cohort 1). The cause of this hemorrhage was determined to be leakage from the retinotomy site both during and after surgery. To minimize this occurrence, the surgical procedure was modified to include several measures: (1) avoidance of any systemic anticoagulants in the perioperative period, including aspirin; (2) diathermy of the retinotomy site if intraoperative bleeding was noted; (3) evacuation of subretinal hemorrhage before and after CPCB-RPE1 implantation; and (4) elevation of intraocular pressure during and after implantation to minimize intraocular hemorrhage from the retinotomy site. These surgical manipulations eliminated any significant perioperative hemorrhage in the subsequently implanted subjects of cohort 2 (Fig. 2).

**Figure 3.**
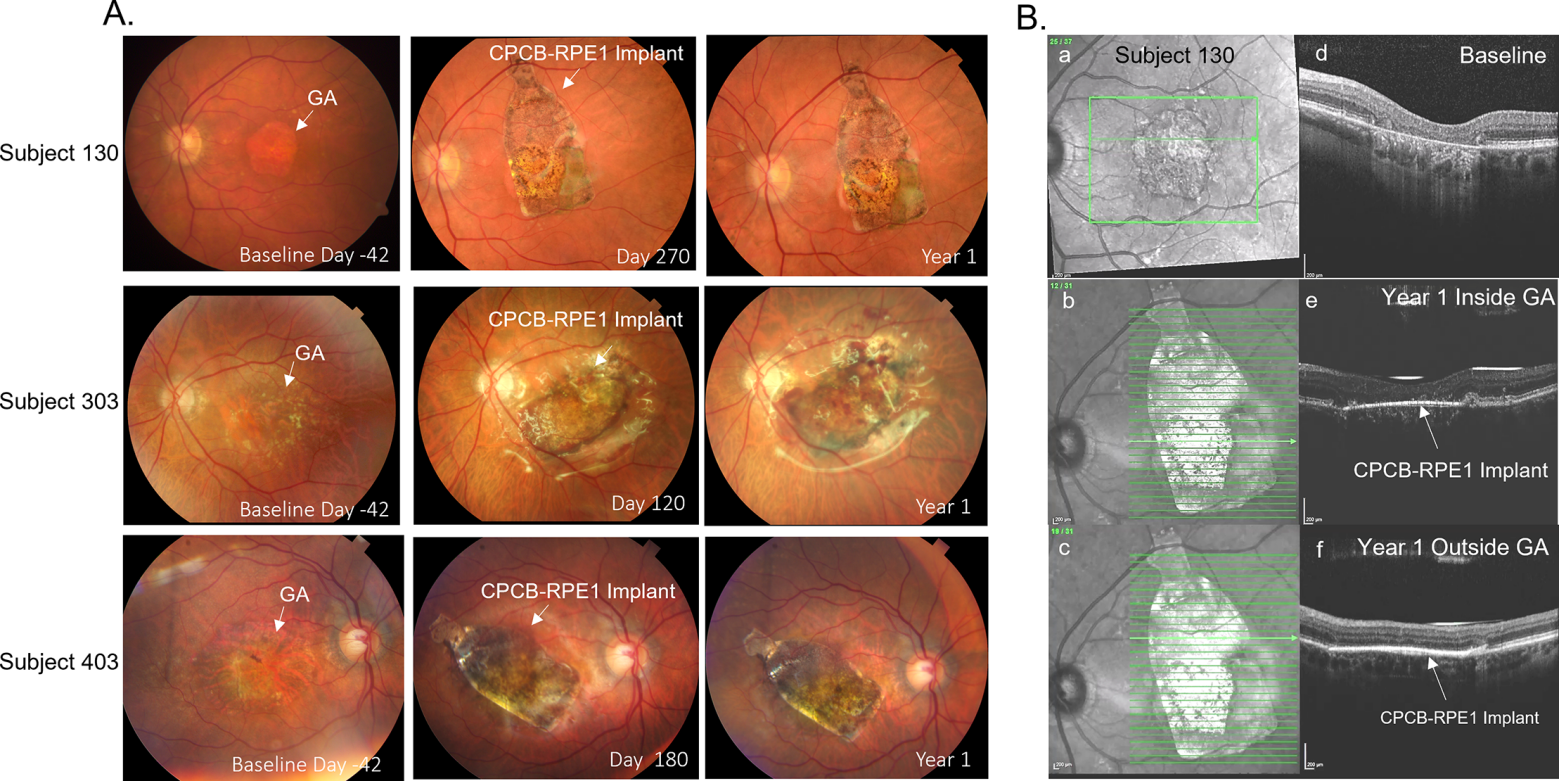
Color fundus photographs and OCT images before and after implantation of CPCB-RPE1. (A) Color fundus photographs from subjects 130 (*top row*), 303 (*middle row*), and 403 (*bottom row*) at baseline, at an intermediate time point, and at 1 year post-implantation. (B) En face OCT images of subject 130 before implantation (a) and at 1 year post-implantation inside (b) and outside (c) the GA lesion. Corresponding B-scans through the area of geographic atrophy prior to implantation (d) and at 1 year post-implantation (e, f) show loss of RPE and photoreceptors prior to treatment. A bright hyperreflective line in the subretinal space indicates the location of the CPCB-RPE1 implant (e, f). The absence of hypertransmission defect in the post-implant images supports survival of transplanted RPE within the region of geographic atrophy.

Figure 3A illustrates preoperative and postoperative 1-year fundus photographs from three representative subjects (subjects 130, 303, and 403). As the figure illustrates, the implant did not move in the subretinal space, and there was no evidence of expanding masses. There was evidence of de-hemoglobinized blood in regions of previous perioperative hemorrhage. Postoperative OCT confirmed the subretinal location of the implant in all cases up to and including day 365 within and outside the area of GA (Figs. 3Bb, 3Bc).

### Adverse Events

Adverse events in the perioperative period associated with the surgery or implant have been previously reported.^14^^,^^16^ Subject 123 underwent surgery but did not receive the implant due to technical problems during surgery that were resolved for all subsequently implanted subjects. Despite not receiving an implant, subject 123 was followed for 1 year for safety, and no ocular serious adverse events were reported; a prolapsed rectum was reported during the year follow-up. For the 15 subjects who received the implant, there were no unexpected serious adverse events associated with the implant, surgery, or immunosuppression through 1 year post-implantation. Table 2 summarizes the serious adverse events during the first year post-implantation. Although silicone oil was instilled in all subjects, in no case was it necessary to remove the silicone oil or to return to the operating room. Four subjects had serious ocular adverse events, including retinal hemorrhage, retinal or macular edema, focal retinal detachment, or RPE detachment. The frequency of these ocular serious adverse events was significantly reduced between cohort 1 and cohort 2 (*P* = 0.02, Fisher's exact test), suggesting that the better hemorrhage control observed in cohort 2 led to reduced ocular serious adverse events. In no case did fluorescein or indocyanine angiography demonstrate choroidal neovascularization. Nevertheless, site investigators were allowed to treat with anti–vascular endothelial growth factor agents at their own discretion. One subject was noted to have a pre-existing peripheral retinal schisis at the time of surgery with possible retinal detachment. The area of schisis and possible retinal detachment was treated with laser barricade without any subsequent sequelae. Notably, there were no reports of vitreous or aqueous cells (after postoperative month 1) and no report at all of vasculitis, choroiditis, or retinitis. In addition, no antibodies to HLA class I or II molecules unique to the implanted RPE cells and not found on the recipients cells were induced in the peripheral blood of the subjects after implantation (manuscript submitted).

**Table 2.** Severe Adverse Events Occurring During the First Year Post-Implantation by System Organ Class and Preferred Term

	*n* (%)		
	Cohort 1 (*n* = 6)	Cohort 2 (*n* = 9)	Total (*n* = 15)
Subjects reporting at least one serious adverse event	6 (100.0)	2 (22.2)	8 (53.3)
Eye disorders	4 (67.7)	0 (0.0)	4 (26.7)^a^
Retinal hemorrhage, deposits, edema	3 (50.0)	0 (0.0)	3 (20.0)
Detachment of retinal pigment epithelium^+^	1 (16.7)	0 (0.0)	1 (6.7)
Macular edema, exudates^b^	1 (16.7)	0 (0.0)	1 (6.7)
Focal retinal detachment^b^	1 (16.7)	0 (0.0)	1 (6.7)
Gastrointestinal disorders	1 (16.7)	1 (11.1)	2 (13.3)
Colitis ischemic	0 (0.0)	1 (11.1)	1 (6.7)
Small intestinal obstruction	1 (16.7)	0 (0.0)	1 (6.7)
Infections and infestations, pneumonia	0 (0.0)	1 (11.1)	1 (6.7)
Cardiac disorders, cardiac failure	1 (16.7)	0 (0.0)	1 (6.7)
Investigations, decreased weight	1 (16.7)	0 (0.0)	1 (6.7)
Neoplasms benign, malignant, and unspecified (including cysts and polyps), esophageal adenocarcinoma	1 (16.7)	0 (0.0)	1 (6.7)

a *P* = 0.02 between cohorts 1 and 2 (Fisher's exact test). *P* values between cohorts 1 and 2 for non-ocular disorders were all nonsignificant.

b Occurred in same subject and in one subject with retinal hemorrhage, deposits, and edema

Severe subject-related systemic adverse events were noted in five subjects. One subject was diagnosed with esophageal adenocarcinoma that was unrelated to the surgery, implant, or immunosuppression associated with this study. Another subject had an exacerbation of congestive heart failure requiring hospitalization which was also not associated with the surgery, implant, or immunosuppression associated with this study. A separate subject was diagnosed with pneumonia requiring hospitalization during the period of immunosuppression taper postoperatively. For this subject, immunosuppression was stopped before the full course was administered; however, there was no evidence that the pneumonia was associated with the surgery or implant. There was no clinical evidence of graft rejection in any subject.

### Visual Acuity

The mean and median BCVA values of the treated eye at day 365 were logMAR 1.33 ± 0.42 and 1.24 (Q1, 1.04; Q3, 1.38), respectively. Although the severity of the baseline GA in the subjects in this study likely precluded significant improvement in visual acuity (all subjects in the study were legally blind), more implanted eyes than non-implanted, contralateral eyes had gained >5 letters in BCVA (27% vs. 7%; *P* = not significant, Fisher's exact test) at day 365. Early Treatment Diabetic Retinopathy Study (ETDRS) letter improvements ranged from 6 to 13 letters at 1 year. Similarly, more implanted eyes than non-implanted eyes had gained >5 letters BCVA or remained within 5 letters of baseline BCVA (67% vs. 53%; *P* = not significant, Fisher's exact test). Fewer implanted eyes than non-implanted, contralateral eyes lost >5 letters of BCVA at 1 year (33% vs. 47%; *P* = not significant, Fisher's exact test).

The percentages of fixation events falling in the central 2° and 4° fields surrounding the preferred retinal locus at baseline were 58.9% ± 34.5% and 87.2% ± 20.5%, respectively (Supplementary Fig. S2). The corresponding values at 365 days (postoperative) follow-up were not significantly different at 53.3% ± 21.6% and 80.5% ± 19.0%. As Supplementary Figure S2 illustrates, there was no significant change in fixation ability in either the implanted or fellow eye over the course of the study.

Most subjects were not able to reliably perform microperimetric sensitivity testing at baseline due to unstable fixation. In most cases, this was due to subject fatigue and the length of time required to perform the assessment. However, one subject who was able and willing to perform this testing at postoperative days 42 through 365 demonstrated both qualitative and quantitative improvement in retinal sensitivity within and surrounding the area of the implant (Supplementary Fig. S3). Specifically, this subject demonstrated a significant improvement in mean retinal sensitivity for all co-registered retinal locations tested at postoperative days 180, 270 and 365 compared with that of postoperative day 42 (baseline data not available). This finding was largely true for retinal loci both within and outside the area of the implant at all time points, with the highest magnitudes of improvement found over the implant. In contrast, there was no significant change in the mean sensitivity of co-registered retinal loci tested in the non-implanted eye (Supplementary Fig. S3).

These visual acuity and visual function data support the safety of the procedure, implant, and immunosuppression at the 1-year time point.

## Discussion

We present the 1-year safety results of 15 subjects implanted in a first-in-human, phase 1/2a study of CPCB-RPE1 for the treatment of severe vision loss and geographic atrophy associated with NNAMD. All subjects were treated on an outpatient basis with one preplanned surgical intervention for subretinal placement of the implant within the area of GA. The surgical procedures were well tolerated, and adverse events associated with surgery were manageable. There were no unanticipated ocular serious adverse events. Furthermore, there was no evidence of implant migration. Preliminary signals of efficacy included improvement of BCVA by >5 letters in 27% of implanted subjects, despite the large extent of retinal degeneration observed in these late-stage subjects. Exploratory data suggest retinal sensitivity is at least maintained over and around the implant. This phase 1/2a study demonstrates the safety of the implant, surgical procedure, and immunosuppression regimen and provides critical insights for future studies aimed at definitively assessing efficacy.

Although the current study demonstrates the safety of the implant, surgery, and immunosuppression, the data also suggest that several improvements made to the surgical procedure during the phase 1/2a study are desirable for future trials. Widely used surgical methods that were implemented successfully minimized intraocular hemorrhage in the perioperative period, and continued implementation of these relatively simple interventions in future studies is warranted. It is likely that they will significantly increase the chances of visual improvement. In addition, the current data suggest that the use of silicone oil is not necessary. Out of an abundance of caution, silicone oil was used in this study as a tamponade, but none of the implants migrated during the first year of the study, and the silicone oil was removed in five and 10 subjects within the first and second year, respectively, of follow-up. Therefore, we plan to avoid the use of silicone oil in future studies, which will significantly reduce the duration of the surgery while reducing a potential stimulus of edema and the need for additional procedures for its removal (see below).

The data from this study also suggest that subjects who received the implant were more likely to improve by >5 letters, and non-implanted eyes were more likely to lose >5 letters. Although the number of subjects in this study does not provide power for a definitive conclusion about efficacy, this trend is encouraging for this advanced stage of the disease treated. Natural history studies of GA demonstrate that patients with a mean ETDRS letter score of 60 letters (20/63) lose an average of 12 letters by 2 years and 23 letters by 5 years.^17^ Therefore, the preliminary efficacy results from this study suggest that the risk–benefit profile of this procedure is acceptable for subjects with progressive vision loss without other treatment options for abrogation of visual decline.

The documented safety of CPCB-RPE1 treatment supports recruitment of subjects with better visual acuity. Subjects with better visual acuity will have a shorter duration of disease, smaller region of GA, and enhanced visual potential attributable to a higher frequency of surviving photoreceptor cells able to benefit from the rehabilitating effects of the implanted RPE cells. Also, preliminary data in this study suggest that subjects exhibiting the most impressive improvement in visual acuity were those with the smallest areas of GA. Finally, our experience indicates that the surgical separation of the retina overlying the area of GA is easier and faster in less chronic cases.

Additional improvements can be implemented in future trials to further improve the adverse event profile. For example, several subjects in the current study had evidence of macular edema not associated with choroidal neovascularization. A possible cause of this postoperative cystoid macular edema is long-term use of silicone oil.^18^ As indicated above, we believe use of silicone oil is unnecessary to control the position of the implant, and avoiding the use of silicone oil will likely improve the adverse event profile and avoid the need for second surgery. Furthermore, although aggressive treatment of cystoid macular edema in well-sighted subjects (e.g., post-cataract surgery) is widely accepted, its treatment is not standard practice in subjects with visual acuity as poor as in those enrolled in this study. Three subjects in the study received intravitreal anti–vascular endothelial growth factor agents at the discretion of the site principal investigators without significant improvement in edema or vision. Therefore, it is very reasonable to aggressively treat evidence of postoperative cystoid macular edema with nonsteroidal and steroidal topical treatments so as to better realize the true visual benefit of this and likely other cell-based therapies for NNAMD.

We believe the mechanism of action for the CPCB-RPE1 is the result of enhanced viability of surviving photoreceptor cells in the retina directly overlying or adjacent to the CPCB-RPE1 implant. This possibility is consistent with the limited evidence we have from microperimetric sensitivity studies of subject 216, but additional data are needed from definitive efficacy studies. It has been demonstrated that visual function in GA subjects is primarily derived from preferred retinal loci at the edge of the GA lesion.^19^ Trophic effects may extend beyond the physical limits of the implant in a manner analogous to those of subretinally injected hESC-RPE suspensions that have been demonstrated to have functional benefit without clear anatomic correlates of RPE–photoreceptor integration.^20^ We also hypothesize that “dormant” photoreceptors are present in the area of GA that are rehabilitated by the CPCB-RPE1 implant and are able to form outer segments. Such “dormant” photoreceptors have been postulated by others in retinal degenerations^20^ and demonstrated on histopathology.^22^

## Supplementary Material

Supplement 1
