# Supplementary material for: One-Year Follow-Up in a Phase 1/2a Clinical Trial of an Allogeneic RPE Cell Bioengineered Implant for Advanced Dry Age-Related Macular Degeneration
Source: Transl Vis Sci Technol. 2021 Oct 6;10(10):13. doi: 10.1167/tvst.10.10.13 (PMC8496407; doi:10.1167/tvst.10.10.13)
Supplement: Supplement 1 [file tvst-10-10-13_s001.pdf]

Supplementary Materials TVST 21-3657

Supplemental Figure 1. Phase 1/2a Clinical Trial Study Schema and Timeline

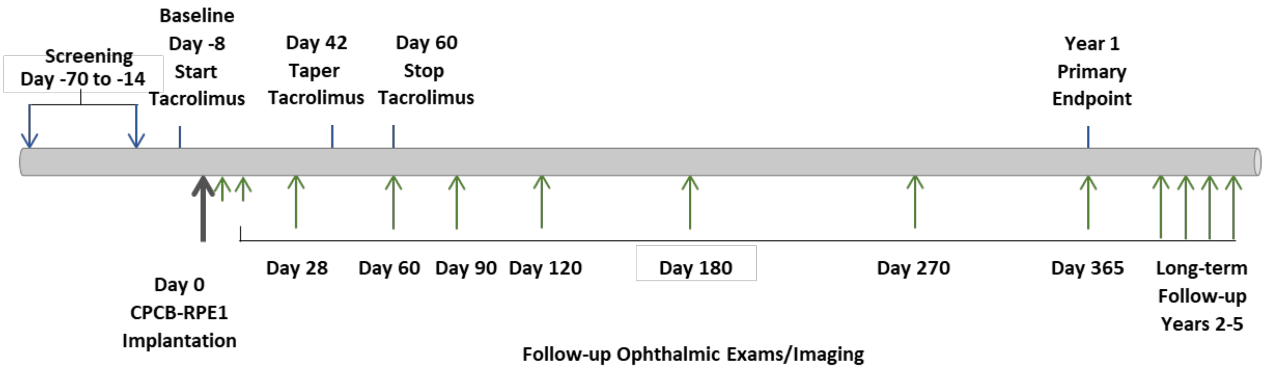

Supplemental Figure 1 Legend

Phase 1/2a Clinical Trial Study Schema and Timeline. Screening evaluations were performed between day -70 and day -14. Low dose Tacrolimus immunosuppression was initiated at day -8 and continued through day 60 with a tapering dose regimen based on regularly assessed serum trough concentrations. The CPCB-RPE1 was implanted on day 0 during a single, outpatient surgery procedure. Postoperative evaluations were performed on days 7, 14, 28, 60, 90, 120, 180, 270 and 365. Long-term, in-person follow-up is being performed annually in years 2-5.

Supplemental Figure 2. Fixation Testing at Baseline and One-Year of Follow-up

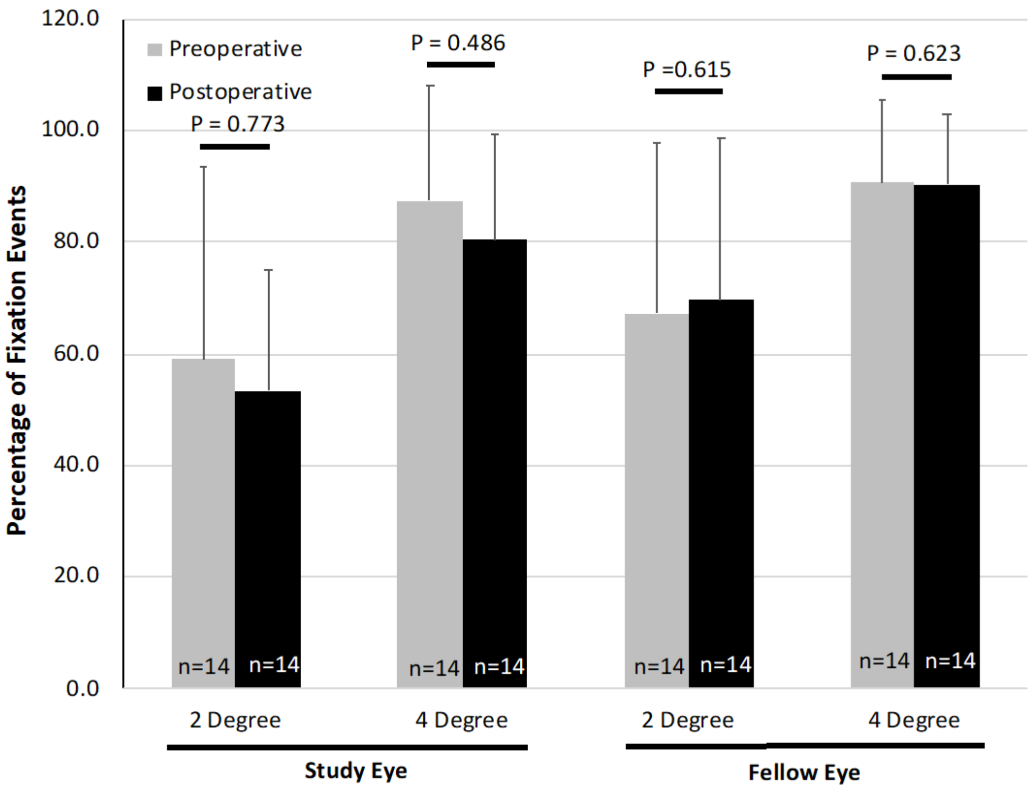

Supplemental Figure 2 Legend

Fixation analyses from testing at baseline (preoperative) and 365 days postoperative follow-up using central 2 and 4 degree regions centered on the preferred retinal locus. Fixation testing was available for N=14 subjects excluding one subject who did not receive the implant and one subject who could not make it to the day 365 study visit to complete fixation testing. There was no statistically significant difference in fixation ability for either the implanted or fellow eye between baseline and 365-day follow-up evaluations.

Supplemental Figure 3. Retinal Sensistivity Data Collected at Day 42, 180, 270 and 365 Follow-up Visits for Subject 216

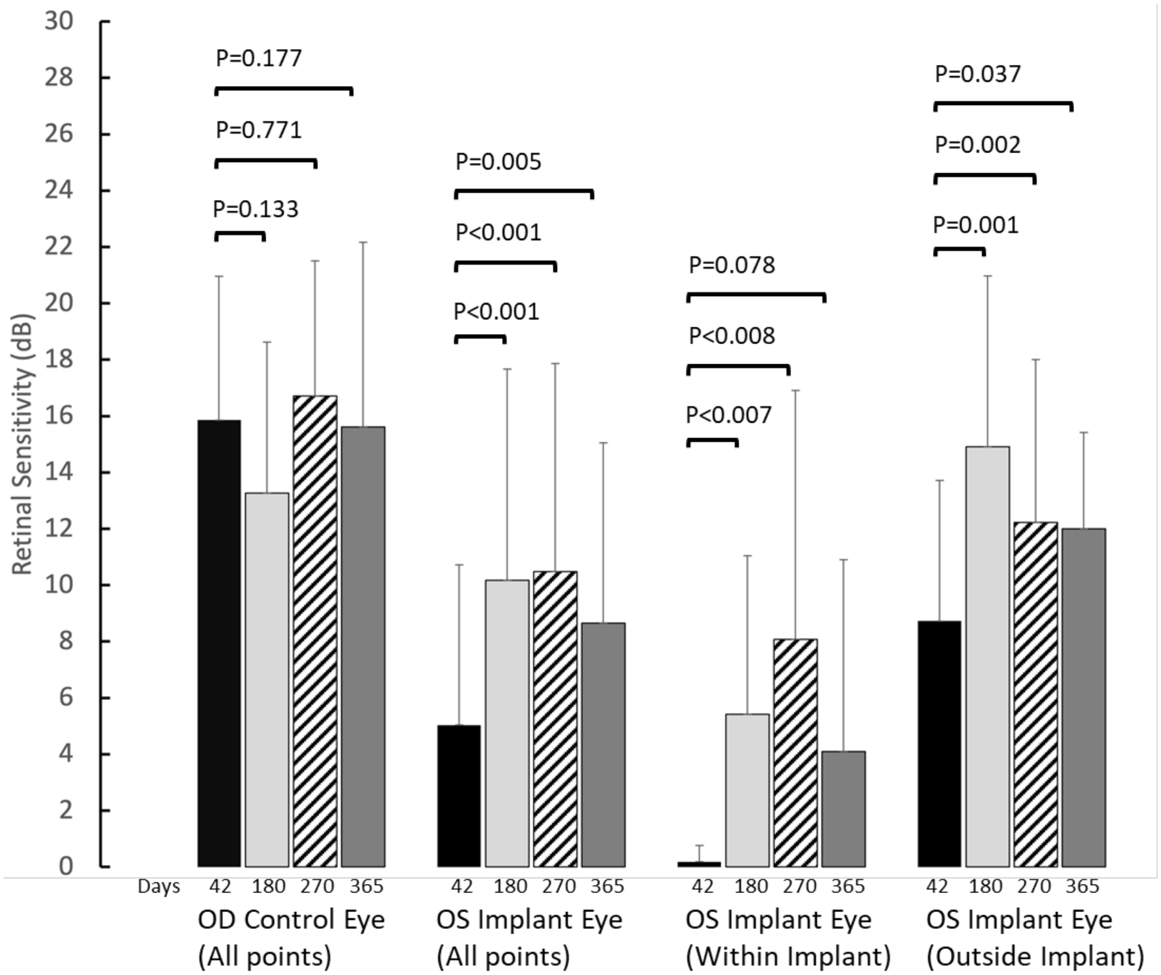

Supplemental Figure 3 Legend.

Microperimetric retinal sensitivity data from subject 216 was available at postoperative days 42, 180, 270 and 365. Mean retinal sensitivity from co-registered retinal loci that were available from all time points in the non-implanted control eye were 15.86±5.10db, 13.27±5.36dB, 16.71±4.80dB, and 15.62±6.55dB (mean +/- SD) at the respective time points. In contrast, mean retinal sensitivity from all co-registered retinal loci that were available from all time points in the implanted eye were 5.04±5.69dB, 10.17±7.50dB, 10.48±7.38dB, 8.65±6.40dB (mean +/- SD) at

the respective time points. Mean retinal sensitivity from co-registered retinal loci located immediately overlying the implant were 0.18±0.59db, 5.41±5.63dB, 8.08±8.82dB, and 4.10±6.81dB (mean +/- SD) at the respective time points. Mean retinal sensitivity from co-registered retinal loci located outside the region of the implant were 8.72±4.99db, 14.92±6.05dB, 12.22±5.79dB, and 12.00±3.42dB (mean +/- SD) at the respective time points. Results of pairwise, two-tailed t-tests between individual time points are illustrated in the figure. Significance was assessed at the Bonferroni corrected p-value of 0.017.
